# Suicide Attempts and Perceived Social Support among Chinese Drug Users: The Mediating Role of Self-Esteem and Depression

**DOI:** 10.3390/ijerph18010208

**Published:** 2020-12-30

**Authors:** Yali Deng, Xuemeng Li, Liu Liu, Wing Hong Chui

**Affiliations:** 1 School of Social Work, University of Maryland Baltimore, Baltimore, MD 21201, USA; yali.deng@ssw.umaryland.edu; 2 The Graduate Center, City University of New York, New York, NY 10016, USA; xli5@gradcenter.cuny.edu; 3 School of Social and Behavioral Sciences, Nanjing University, Nanjing 210023, China; 4 Department of Social and Behavioural Sciences, City University of Hong Kong, Hong Kong, China; eric.chui@cityu.edu.hk

**Keywords:** suicide attempts, perceived social support, depression, self-esteem, Chinese drug users

## Abstract

Suicidal behavior is a severe problem among drug users. This study examines influential factors related to suicide attempts and analyzes possible mediators of the relationship between perceived social support and suicide attempts amongst Chinese drug users under compulsory institutional drug treatment. Taking perceived social support as the independent variable, we found that the relationship between suicide attempts and perceived social support is mediated by self-esteem as a protective factor and depression as a risk factor. Path analysis shows that self-esteem contributes relatively more to the indirect effects than depression does, accounting for 31.1% and 24.2% of the total effect, respectively. Generally speaking, the findings of this study point to an urgent need for addressing suicide attempts among Chinese drug users while treating self-esteem as the protective factor that deserves as substantial attention as depression receives.

## 1. Introduction

Suicide is a worldwide health problem. According to the World Health Organization [1], in 2016, the global suicide rate was 10.5 per 100,000 people, and suicide is the second leading cause of death among people aged 15 to 29. In China, the suicide rate was 8.0 per 100,000 people in 2016. While the suicide rate in China was lower than that of the global rate, the number of suicide deaths was still high, namely 136,267, accounting for about 17% of the world’s suicide deaths (800,000 per year) [1]. In China, the national suicide rates decreased significantly from 2002 to 2015. However, the drop in suicide rates slowed down around 2007, and the rate even increased in some subgroups, such as young male adults [2]. Male and rural populations have a higher suicide rate and a lower decreasing trend than female and urban populations [3]. Chinese studies found similar predictors of suicide to those in other cultures, including physical health, psychological and psychopathological factors, social support, and stressful life events [4].

Drug users are primarily documented as a high-risk population of suicidal behavior [5,6], since drug use can exert a negative impact on individuals’ health and mental health, and can also cause problems such as cognitive neurological disorder, depression, and anxiety [7,8]. For example, previous research findings reported that the occurrence of suicidal ideation and suicide attempts among illicit drug users were as high as 55% and 32%, respectively [9,10]. Moreover, the suicide mortality rate of heroin users was found to be 16.2 fold higher than that of the general population [11].

Suicide issues among drug users have become salient in China, considering its large population of drug users. Along with its rapid economic growth, the Chinese society has been facing a fast and steady increase in the drug use rate since the late 1970s [12,13]. The number of Chinese registered drug users, i.e., those caught by police at least once for drug use, has doubled in the past decade and grew to 2.5 million in 2016 [13]. While China has seen a decrease in registered drug users from 2017 to 2019, the total population remains large, i.e., 2.14 million by the end of 2019 [14]. Previous empirical studies in China showed that the rates of lifetime suicidal ideation and suicide attempts among methadone-maintained patients were 58.9% and 34.2%, respectively [15,16]. The rate of suicidal ideation among male and female drug users under compulsory institutional drug treatment was 31.5% and 21.3%, respectively [17,18]. The rate of suicidal ideation after enrolling in compulsory treatment facilities was higher than pre-treatment rates (24.6% vs. 16.4%) [19]. Under such a context, the studies on Chinese drug users’ suicidal behavior are much needed.

## 2. Literature Review and Theoretical Perspective

“Suicide” used in the introduction section refers to completed suicide. In fact, the nomenclature of “suicidology” falls into three distinct categories―suicidal ideation, suicide attempt, and completed suicide [20,21]. Suicidal ideation refers to thoughts of ending one’s own life [22]. Suicide attempt as a behavioral consequence of suicidal ideation [23] refers to self-harm behavior with a certain degree of suicidal ideation and a non-fatal result [24]. The third terminology, completed suicide, is used when the outcomes are fatal [25]. Logically, these three distinct terms, suicidal ideation, suicide attempt, and completed suicide, form a progression from ideation to action, whilst illustrating three stages of suicidality with different severity of consequences [26]. This study uses suicide attempt as the dependent variable, rather than suicidal ideation or completed suicide, because suicide attempt connects suicide ideation and completed suicide. Suicidal ideation does not inevitably lead people to commit suicide [27]; however, suicide attempts have been found to be the most significant predictor of the next attempt and completed suicide [28,29]. Moreover, assessing suicide attempts is more direct and reliable than assessing suicidal ideation. Suicidal ideation is a subjective thought or latent variable that cannot be observed and is thus hard to precisely measure [30]. In contrast, suicide attempt as a concrete behavior is observable and countable. Compared to the data collection of completed suicide, which often requires interviews with the decedents’ family members or close relatives [31], suicide attempts could be self-reported.

Among studies of suicide, many traditional theories focused on the risk factors, which refer to the influencing factors that encourage the occurrence of a negative result [32], such as psychache [33], burdensomeness, and social alienation [34]. Klonsky and May [27] proposed a three-step theory of suicide, which incorporates connectedness as a protective factor against the escalation of suicidal ideation. Connectedness is defined broadly, referring to the connection of other people or “any sense of perceived purpose or meaning that keeps one invested in living” [27] (p. 117).

Social support as a kind of connectedness is documented to be a protective factor of lifetime suicide attempts [35]. Social support refers to an asset, including instrumental, emotional, informational resources, and social companionship that one can obtain from their social networks [36]. Social support can be qualitative and quantitative [37], and can be further divided into received social support and perceived social support [38]. Received social support is defined as the morphological properties of individuals’ social networks, measured quantitatively through the networks’ reachability, density, anchorage, and range [39]. Perceived social support refers to individuals’ perception or general satisfaction of the support from others in their social network [40]. Compared to received social support, perceived social support as a subjective feeling has been proved to have a more significant effect on one’s mental health [41], and mental health is highly related to suicidal behavior among drug users [9]. For example, a higher likelihood of suicide attempts was found to be associated with a lower level of perceived parental support for adolescents [42]. In this regard, the present study used perceived social support as the independent variable, rather than received social support.

Defined as people’s perception of themselves [43], self-esteem is also found to act as a protective factor of suicidal behavior [44]. According to a study with substance users, those with higher self-esteem have a lower risk of suicidal ideation [45]. Previous studies have also proved that self-esteem is a potential factor that mediates the relationship between perceived social support and suicidal ideation [46]. For example, Kleiman and Riskind [46] revealed that perceived social support might increase self-esteem and then reduces the strength of suicidal ideation among college students. However, its mediating effect among drug users in the suicide attempts/perceived social support relationship remains unclear.

In addition to protective factors, there are also risk factors for suicide attempts. Psychological distress and mental disorders were risk factors of suicide attempts among drug users, including depression, anxiety, borderline personality disorder, and post-traumatic stress disorder [11,47,48,49]. Among all the risk factors, this study explores the possible mediating function of depression for the association between suicide attempts and perceived social support. Studies on rural left-behind children have shown that depression could be a mediator to the relationship between suicidal ideation and perceived social support [26]. In other words, depression is not only directly related to suicidal ideation [50] and perceived social support [51], but also standing in between, serving a mediating role for the relationship [52]. This study intends to swap suicidal ideation with suicide attempts and examine the possible associations with the sample of Chinese drug users, an underexplored group.

Suicide attempts are also found to be associated with some demographic factors among Chinese drug users, including gender, education level, and age. Evidence has shown that a high likelihood of suicide attempts is related to females with an educational level of primary school or lower and younger age (20–39 years old) among Chinese individuals receiving methadone maintenance treatment [16,53]. However, another study conducted in Taiwan has found that recent suicide attempt is associated with a higher level of education [54], and studies in some western societies found that female heroin users have higher rates of suicidal ideation and attempts while having lower rates of completed suicide than male heroin users [9]. Further, being single/divorced/widowed [16], having a long history of drug use [17], and experiencing adverse childhood experiences [55] may also increase the chance of suicide attempts among drug users. As a result, these factors were used in the analytical models as control variables.

In summary, although the studies on Chinese drug epidemics have grown substantially in recent years, research on drug users’ suicide attempts is minimal. To fill this gap, we intend to provide newer empirical data for suicide attempts and explore the influencing factors among Chinese drug users. While there have been numerous studies on suicidal ideation and perceived social support, the relationship between suicide attempts and perceived social support remains underexplored. Additionally, our research aims to explore the possible mediators for the association, primarily self-esteem and depression. Thus, we propose the following hypotheses: (1) Suicide attempts are negatively associated with perceived social support. (2) Suicide attempts are negatively associated with self-esteem. (3) Suicide attempts are positively associated with depression. (4) Self-esteem and depression mediate the effect of suicide attempts on perceived social support.

## 3. Materials and Methods

### 3.1. Participants and Procedures

This study is based on a cross-sectional survey conducted in nine compulsory drug treatment institutions in China. China has a “zero-tolerance” policy on using illicit drugs, including “opium, heroin, methamphetamine, morphine, marijuana, cocaine, 121 types of narcotic drugs, and 130 types of psychotropic drugs” [56] (p. 310). Individuals caught using the aforementioned illicit drugs would face administrative punishment. They would be fined for being caught for the first time or required to participate in a community-based treatment program if caught for the second time. Being caught for the third time or more would make them subject to a two-year compulsory institutional drug treatment [57,58], which involves vocational training and educational rehabilitation, but does not involve any psychiatric drug treatment [57]. In 2016, 357,000 drug users were subjected to institutional compulsory drug treatment, accounting for the largest group of drug users who received treatment [59]. The research protocol was approved by the School of Social and Behavioral Sciences of Nanjing University to make sure that steps were taken to protect human research participants, and the research was conducted in an ethical manner.

Stratified cluster sampling was used to recruit participants. Two residential halls were randomly selected from each of the nine compulsory drug treatment institutions. All individuals (around 200) in each selected residential hall were invited to complete the self-administered questionnaires. Research participants were fully informed of this study’s purpose, their voluntary participation, and the obligation of researchers to protect the privacy of participants and maintain the confidentiality of data before completing the questionnaire. The trained research assistants also emphasized that, if prospective participants declined participation, it would not affect their continued access to drug treatment programs—no penalties would be imposed for their decline. A total of 3473 questionnaires were distributed, and 3239 valid responses (70% male and 30% female) were received with a response rate of 93.3%. Among them, 27.5% were meth users, i.e., people who primarily used meth prior to residential treatment; 13.2% were heroin users, and 2.1% used other drugs such as cocaine, ecstasy, ketamine, and marijuana. About 57.2% of the participants refused to report the type of drug they primarily used.

### 3.2. Variables and Measures

All the valid samples from the survey were used for the logistic regression and path analyses, though listwise deletion was applied to variables with missing values. The final sample consisted of 1895 Chinese drug users. Suicide attempt, as the dependent variable, was measured by a “yes” or “no” single-choice question, “Have you ever attempted suicide in the past 30 days?” (Yes = 1; No = 0). Considering perceived social support is a latent variable that is hard to observe, a well-developed scale, the Multidimensional Scale of Perceived Social Support (MSPSS), was used to measure the concept. With MSPSS, drug users’ perceived social support from family, friends, and significant others could be measured [60]. MSPSS contains 12 items, and a 7-point Likert scale measures each item (1 = Strongly Disagree and 7 = Strongly Agree). In this study, scores for all items were summed up to compute a total score to predict the perceived social support of each case. A higher score indicates a higher level of perceived social support. This scale has been proven to have high reliability and validity by various psychometric evaluation scales across different academic disciplines [60,61]. In this study, the Cronbach’s α of the MSPSS was 0.87.

We hypothesize that self-esteem and depression to be the mediators of suicide attempts regress on perceived social support. Self-esteem was measured by Rosenberg’s Self-Esteem Scale (SES) [43], a 10-item scale with some positive and some negative dimensions. This scale was rated by a 4-point Likert scale (1 = Strongly Disagree, 2 = Disagree, 3 = Agree, 4 = Strongly Agree). The score of the negative domain was reversed, and then the scores for ten items were summed up to compute a total score. The higher the score, the higher level of self-esteem. The Chinese version of SES has been proved reliable among Chinese drug users [62]. The Cronbach’s α of SES in this study is 0.77.

The severity of depression was measured by Zung Self-Rating Depression Scale (SDS) [63]. This scale contains 20 items, 10 items assessing the positive domain and 10 items assessing the negative domain. All items were rated by a 4-point Likert scale ranging from 1 (Rarely) to 4 (Almost Always). We reversed the score of the positive domain and summed up the rating of all items. A higher total score indicates a higher level of depression. The Chinese version of SDS has been employed in various populations and has shown great reliability and validity [64]. In this study, the Cronbach’s α of SDS is 0.70.

Finally, control variables include demographic factors like gender, age, education level, marital status, length of drug use, and abuse experience. The dichotomous variables, gender, marital status, and abuse experience, were coded as dummy variables. Marital status was recoded as “single (including divorced and widowed) = 0” and “married (including cohabitation) = 1”. History of abuse was measured by two-option “yes” or “no” single-choice questions, “Have you ever experienced physical abuse during your lifetime” and “Have you ever experienced emotional abuse during your lifetime”. A positive answer to either question indicates a history of being abused, while negative responses for both indicate no history of being abused. The non-dichotomous categorical variable, level of education, was recoded as “primary school or below = 1”, “middle school = 2”, “high school = 3”, and “junior college or above = 4”. Continuous variables―age and length of drug use variables—were used in our analysis in the same form as the data was initially collected.

### 3.3. Analytical Process

Binary logistic regression was applied to examine factors associated with suicide attempts because the dependent variable is a dichotomous variable, namely had or never had suicide attempts. Moreover, logistic regression is a function of the logarithm of the ratio between the probabilities of something happening and not happening [65]. Therefore, it is usually used to predict the likelihood of an event. In this study, we ran five different models with logistic regression to provide an exploratory description of the associations between the dependent variable, independent variable, control variables, and the two primary mediators. In Model 1, the dependent variable regresses only on the independent variable, while in Model 2, control variables were added. In Models 3 and 4, self-esteem and depression were added, respectively, on top of Model 2. Finally, we put all variables in Model 5.

Furthermore, path analysis with logistic regression was performed to examine the primary mediating role of self-esteem and depression, both respectively and combined. The KHB method developed by Karlson, Holm, and Breen [66] was applied in path analysis with Stata 14. As a general decomposition method, the KHB method is used for “comparing the estimated coefficients of two nested nonlinear probability models” [66] (p. 420). First, the KHB method decomposes the total effect into direct and indirect effects and then further produces a confounding percentage that interprets the magnitude of the indirect effect. Second, the KHB command in Stata allows more than one mediator in one path analysis and disentangles the contributions of these mediators. Thus, we can compare the confounding percentages to determine one of the multiple mediators contributing most to the confounding. In this study, three mediation analyses with two different possible mediators were conducted. We firstly examined the probable mediation effect of self-esteem and depression individually and then put them together into the mediation model for path analysis to compare their contributions to the indirect effect. We only focused on the dependent variable, independent variable, and two mediators; thus, no controls were included in all three path analyses.

## 4. Results

### 4.1. Description of Samples

After list-wise deletion, we first performed descriptive statistics on all variables. As all continuous variables have a skewness between −1 and 2, we view distributions as normal and report mean values for their central tendency. For the dependent variable, 7.8% of the respondents had suicide attempts in the last 30 days (Table 1). Table 1 presents the mean value and standard deviation of perceived social support, self-esteem, and depression; perceived social support shows the highest dispersion. Male drug users were the majority (70%) of all participants. Nearly two-thirds (63.2%) of drug users only completed nine years of education—middle school or less. Approximately 43.6% of the respondents were married, including cohabitation. The data showed that 22.2% of the respondents experienced physical or emotional abuse during their lifetime. The average age was 32.5 years old, while the average length of drug use was close to ten years.

In Table 2, the correlation matrix presents correlations and the possible collinearity between all continuous variables, namely perceived social support, self-esteem, depression, age, and length of drug use. All correlation coefficients were under 0.7. Perceived social support, self-esteem, and depression were all significantly correlated with one another. A higher level of perceived social support was correlated with a higher level of self-esteem (r = 0.188) and a lower level of depression (r = −0.141); a higher level of self-esteem indicates a lower level of depression, and vice versa (r = −0.391). Age was positively correlated with the length of drug use (r = 0.62), inferring a certain level of lifetime drug use behavior.

### 4.2. Logistic Regression for Suicide Attempts on Perceived Social Support

Binary logistic regression analysis with five different models was used to explore the associations between suicide attempts and perceived social support, controlling for demographic variables, and possible mediators, self-esteem, and depression (Table 3). Model 1 examines the possible association between suicide attempts and perceived social support. Suicide attempts have been found to be negatively associated with perceived social support. For every one-unit increase in perceived social support, we expect a 2% (e^−0.014^−1, *p* < 0.05) decrease in the likelihood of suicide attempts. Model 2 has control variables added. The significance of the association between suicide attempts and perceived social support disappears, while suicide attempts were positively associated with male and abuse experience. Compared to females, male drug users have 125% (e^0.812^−1, *p* < 0.001) higher likelihood of attempting suicides holding all other variables in the model constant. Drug users in the study with abuse experience have 77% (e^0.573^−1, *p* < 0.01) higher likelihood of attempting suicides compared to those who had never experienced abuse.

In Model 3, we added self-esteem on top of Model 2, and suicide attempts were negatively associated with self-esteem. For every one-unit increase in self-esteem, the likelihood of suicide attempts decreases by 10% (e^−0.109^−1, *p* < 0.001), holding all controls constant. In Model 4, we swapped self-esteem with depression in Model 3, and suicide attempts were positively associated with depression. For every one-unit increase in depression, the likelihood of suicide attempts increases by 6% (e^0.057^−1, *p* < 0.001), holding all controls constant. Finally, all variables were put into Model 5. Suicide attempts were still significantly associated with self-esteem, depression, male, and history of abuse.

In summary, suicide attempts were only significantly associated with perceived social support in Model 1. When controls were added, the association between suicide attempts and perceived social support disappears, while other factors showed significance. This pattern remained when we added self-esteem and depression, both individually and combined in Models 3–5. This result implies not only a possible spurious association between suicide attempts and perceived social support, but also potential mediators. Therefore, mediation analyses were performed.

### 4.3. Mediation Analyses

The KHB method is applied to examine the mediating effect of self-esteem and depression on the relationship between suicide attempts and perceived social support. We performed three mediation analyses, including the dependent variable, independent variable, and mediators; no control variable was added (Table 4).

Firstly, we took self-esteem as the only mediator. The effect of perceived social support, when mediated by self-esteem, reduced from 0.015 to 0.008, leaving an indirect effect of 0.007; in other words, 45.2% of the total effect is contributed by self-esteem.

Secondly, we took depression as the only mediator in the relationship between suicide attempts and perceived social support. The effect of perceived social support, when mediated by depression, reduced from 0.015 to 0.01, leaving an indirect effect of 0.005; in other words, 33.5% of the total effect is due to depression.

Finally, we took both self-esteem and depression together into the mediation model. These two variables reduced the effect of perceived social support from 0.016 to 0.007, with an indirect effect of 0.009, accounting for 55.3%. Compared to depression (24.2%), self-esteem (31.1%) contributed 6.9% more to the indirect effect.

## 5. Discussion

In this study, we explored suicide attempts and associated factors among Chinese drug users under compulsory institutional drug treatment, and further investigated the mediating effect of self-esteem and depression on the relationship between suicide attempts and perceived social support. First, the prevalence of suicide attempts in the last 30 days is 7.8%, which is relatively lower than the numbers reported in previous studies on Chinese drug users, for instance, 9.5% or 10.9% among methadone-maintained patients [16,54]. Additionally, the findings showed that drug users with abuse experience have a significantly higher number of suicide attempts, which is consistent with previous studies [55]. People tend to develop suicidal capacity due to being exposed to abuse [27]. Further, we performed logistic regression analysis and examined the primary mediating role of self-esteem and depression. Being consistent with what we hypothesized, a higher likelihood of suicide attempts was significantly associated with a lower level of self-esteem, and a higher level of depression. We also found that suicide attempts among drug users are not directly affected by one’s level of perceived social support; instead, perceived social support plays its protective role via self-esteem and depression. This finding challenges Hypothesis 1, but confirms Hypothesis 4. In other words, a higher perceived social support was associated with a lower level of depression, which in turn reduces the chance of suicide attempts; meanwhile, perceived social support may reduce the likelihood of suicide attempts by increasing self-esteem.

Firstly, the findings of this study challenge the conclusion from previous studies. We argue that perceived social support has no significant direct effect on suicide attempts [35]. To further explain this finding, we refer to the three-step theory of suicide [27]. There are three steps before reaching the stage of suicide attempts: the emerging of suicidal ideation, the escalating of suicidal ideation, and lastly, suicide attempts and suicidal ideation is bridged by suicide ability. In this theory, loss of social connectedness does not necessarily contribute to the emerging of suicidal ideation, while strong connectedness plays a protective role against the escalating of suicidal ideation. Thus, we believe that perceived social support as a kind of connectedness acts indirectly on suicide attempts by reducing factors that escalate suicidal ideation.

Secondly, the finding of the mediating role of self-esteem between suicide attempts and perceived social support could be explained by the theory of positive thinking [67]. Studies showed that self-esteem could promote one’s positive thinking, which, in turn, enhances their suicide resilience. Apart from self-esteem, depression is also found to mediate the relationship between suicide attempts and perceived social support by our study among drug users. This finding can also be explained by the aforementioned three-step theory of suicide, in which depression, as the most significant risk factor of suicidal behavior [68], enhances the suicidal ideation that brings people to the last stage—suicide attempts. Thus, perceived social support affects drug users’ suicidal ideation through reducing the level of depression, and eventually decreases a drug users’ likelihood of suicide attempts.

Thirdly, using the KHB command, we found that self-esteem as a mediator contributed more amount to the indirect effect than depression did. In other words, self-esteem needs substantial attention in studying suicidal behavior, especially among drug users. Depression is a widely discussed mental health problem in suicide [9], but self-esteem is not. Drug-dependents, in general, experience significantly lower self-esteem than the general population [69]. A qualitative study has indicated that drug use brings a feeling of guilt or self-hate for users, which destroys their self-esteem and self-image [70]. While our study reinforces the importance of depression that has been highlighted in previous studies, we argue that for special populations like drug users, self-esteem could also be an essential protective factor of suicidal behavior.

These findings have significant theoretical and practical contributions. To the best of our knowledge, supported by the three-step theory of suicide, this study is the first to explore the mediating effect of self-esteem and depression on the association between suicide attempts and perceived social support among Chinese drug users. Practice-wise, the finding of this study indicates the importance of developing mental health counseling programs to reduce suicide attempts among drug users, especially those under compulsory institutional drug treatment. They have limited connection with people from outside due to the residential treatment policy and confined environment. The lower level of perceived social support may increase suicide attempts by influencing their mental health conditions. Therefore, programs addressing depression and improving self-esteem would help prevent suicide attempts within the compulsory drug treatment institutions. Further studies can also examine the operation processes and the effectiveness of these programs, thus contributing to drug treatment improvement.

Despite the aforementioned implication, this study has a few limitations. First, the representativeness of the sample is limited. Since all participants were recruited from compulsory institutional treatment programs, the interpretation of the results should be limited to this specific period and situated environment. However, this limitation also enhanced the uniqueness of our empirical data and related findings. Another limitation is the measurement of perceived social support. The scale of MSPSS can only measure people’s general perception, without considering the positive or negative domain of the social support they perceived. However, in the field of criminology, previous studies suggested asking what the social relations’ views on drug users’ deviant behavior [12] are because social support may not always have a beneficial effect. For example, studies showed that having a first-degree relative with a history of suicide attempts was a predictor of suicidal behavior among drug users [49]. Therefore, future research can further consider perceived both positive social support and negative social support. Lastly, this study primarily focused on the psychological and social relationship factors while failing to include physical health factors [71]. Further studies can explore physical health factors, such as perceived health status and sleep disorders [47,72].

## 6. Conclusions

In conclusion, this research sampled Chinese drug users under compulsory treatments, studied the association between suicide attempts and perceived social support, and explored the possible mediating roles of self-esteem and depression. We found that suicide attempts and perceived social support could be a spurious association. Further analysis showed that perceived social support did not directly affect drug users’ suicide attempts but functioned indirectly by reducing the level of depression and improving the level of self-esteem. According to the results of path analysis with the KHB command, we found that self-esteem as a mediator contributes more to the indirect effect than depression does. Our finding also indicates that self-esteem deserves substantial attention in the field of suicide research, especially among drug users.

## Figures and Tables

**Table 1:** Descriptive statistics.

Variable	Mean	SD
Suicide attempts (1 = yes) ^a,b^	0.078	-
Perceived social support	61.4	11.9
Self-esteem	26.7	4.0
Depression	48.4	8.3
Gender (1 = male)	0.70	-
Age	32.5	8.0
Education level		
Primary or below	0.198	-
Middle school	0.434	-
High school	0.267	-
Junior college or above	0.101	-
Marital status (1 = married) ^c^	0.436	-
Length of drug use	9.2	6.6
Ever experienced abuse (1 = yes)	0.222	-

^a^ The number of valid cases is 1895. ^b^ For dummy variables, percentages for category “1” are reported in the cell. ^c^ Married includes cohabitation.

**Table 2:** Correlation between continuous variables.

	PSS ^a^	Self-esteem	Depression	Age	LDU ^b^
PSS	1				
Self-esteem	0.188 ***	1			
Depression	−0.141 ***	−0.391 ***	1		
Age	−0.022	−0.035	0.0143	1	
LDU	0.011	−0.008	0.018	0.621 ***	1

^a^ Abbrevation PSS = perceived social support ^b^ LDU = length of drug use, *** *p* < 0.001.

**Table 3:** Logistic regression models for suicide attempts.

Variables	Model 1	Model 2	Model 3	Model 4	Model 5
Perceived social support	−0.014 *	−0.010	−0.005	−0.006	−0.004
	(0.007)	(0.007)	(0.007)	(0.007)	(0.007)
Self-esteem		-	−0.109 ***	-	−0.075 **
		-	(0.025)	-	(0.026)
Depression		-	-	0.057 ***	0.044 ***
		-	-	(0.012)	(0.013)
Male		0.812 ***	0.800 ***	0.943 ***	0.905 ***
		(0.232)	(0.233)	(0.235)	(0.236)
Age		−0.008	−0.008	−0.009	−0.009
		(0.014)	(0.014)	(0.014)	(0.014)
Education level		0.092	0.115	0.044	0.070
		(0.094)	(0.094)	(0.095)	(0.095)
Being married		0.039	0.045	0.038	0.042
		(0.177)	(0.178)	(0.178)	(0.179)
Length of drug use		0.002	0.002	0.001	0.000
		(0.018)	(0.018)	(0.018)	(0.018)
Ever experienced abuse		0.573 **	0.542 **	0.494 *	0.489 *
		(0.191)	(0.193)	(0.193)	(0.194)
Constant	−1.633 ***	−2.613 ***	−0.138	−5.598 ***	−3.203 **
	(0.412)	(0.630)	(0.837)	(0.896)	(1.218)
Observations	1895	1895	1895	1895	1895
Significance of models	0.043	0.000	0.000	0.000	0.000
Pseudo R-squared	0.004	0.027	0.046	0.050	0.059

Standard errors in parentheses * *p* < 0.05, ** *p* < 0.01, *** *p* < 0.001.

**Table 4:** Self-esteem and depression as mediators among the relationship between suicide attempts and perceived social support.

Mediator	Effect	OR	*p*-Value	% Mediated
Self-esteem	Total	0.985	0.030	45.2
	Direct	0.992	0.239	
	Indirect	0.993	0.000	
Depression	Total	0.985	0.029	33.5
	Direct	0.990	0.148	
	Indirect	0.995	0.000	
All mediators	Total	0.984	0.025	55.3 = 31.1 (SES)
(Self-esteem	Direct	0.993	0.323	+24.2 (SDS)
+ Depression)	Indirect	0.991	0.000	

## Data Availability

The data presented in this study are available on request from the corresponding author. The data are not publicly available due to the privacy.
